# Who is distressed? A comparison of psychosocial stress in pregnancy across seven ethnicities

**DOI:** 10.1186/s12884-016-1015-8

**Published:** 2016-08-11

**Authors:** Alexandra M. Robinson, Karen M. Benzies, Sharon L. Cairns, Tak Fung, Suzanne C. Tough

**Affiliations:** 1 Faculty of Education, Counselling Psychology, University of Calgary, Calgary, AB Canada; 2 Faculty of Nursing, and Department of Paediatrics, Cumming School of Medicine, University of Calgary, Calgary, AB Canada; 3 Information Technologies, University of Calgary, Calgary, AB Canada; 4 Departments of Paediatrics and Community Health Sciences, Cumming School of Medicine, Calgary, AB Canada

**Keywords:** Pregnancy, Mental health, Immigration, Psychosocial stress, Minority status

## Abstract

**Background:**

Calgary, Alberta has the fourth highest immigrant population in Canada and ethnic minorities comprise 28 % of its total population. Previous studies have found correlations between minority status and poor pregnancy outcomes. One explanation for this phenomenon is that minority status increases the levels of stress experienced during pregnancy. The aim of the present study was to identify specific types of maternal psychosocial stress experienced by women of an ethnic minority (Asian, Arab, Other Asian, African, First Nations and Latin American).

**Methods:**

A secondary analysis of variables that may contribute to maternal psychosocial stress was conducted using data from the All Our Babies prospective pregnancy cohort (*N =* 3,552) where questionnaires were completed at < 24 weeks of gestation and between 34 and 36 weeks of gestation. Questionnaires included standardized measures of perceived stress, anxiety, depression, physical and emotional health, and social support. Socio-demographic data included immigration status, language proficiency in English, ethnicity, age, and socio-economic status.

**Results:**

Findings from this study indicate that women who identify with an ethnic minority were more likely to report symptoms of depression, anxiety, inadequate social support, and problems with emotional and physical health during pregnancy than women who identified with the White reference group.

**Conclusions:**

This study has identified that women of an ethic minority experience greater psychosocial stress in pregnancy compared to the White reference group.

## Background

It is well established in health research that ethnic minorities have poorer physical and mental health trajectories than those from the dominant culture [1–5]. This phenomena has also been documented in pregnancy health outcomes [6–10]. Interestingly, the longer a woman who has immigrated lives in Canada prior to pregnancy, the greater her risk for poor pregnancy outcomes [11]. One explanation for this phenomena is that women of an ethnic minority may experience elevated levels of psychosocial stress during pregnancy [12–16]. Psychosocial stressors include psychological, social, emotional, physical, and cultural factors that challenge an individual’s ability to cope [17, 18]. Psychosocial stressors associated with being from an ethnic minority endure throughout the course of pregnancy and motherhood [19] and likely contribute to the deteriorating physical and mental health outcomes of ethnic minorities [7, 19].

A recent longitudinal study of foreign-born Canadian mothers conducted by Zelkowitz and colleagues [11] found that 38 % of pregnant immigrant women surveyed (*n* = 119) met the criteria for Postpartum Depression (PPD) based on the Edinburgh Postnatal Depression Scale [20] two months post parturition. Depressive symptoms in pregnancy can have serious impacts on pregnancy and birth outcomes. In a meta-analysis of health risks of women who experience depressive symptoms in pregnancy, Grote and colleagues [21] identified women of minority status to be at greater risk for depression in pregnancy. The aforementioned study also found that women who reported symptoms of depression during pregnancy were at a greater risk for preterm birth, and their infants had greater risk of lower birth weight and intrauterine growth restriction.

Pregnant women are at an increased risk to develop anxiety disorders [22, 23]. Women of an ethnic minority are reported to experience elevated levels of anxiety in pregnancy compared to their native-born conterparts [24]. The experience of state anxiety in pregnancy has been correlated with increased risk of preterm birth [25], low birth weight [26], intrauterine growth restriction [27], neurodevelopmental problems [28], childhood behavioural problems [29, 30], and postnatal depressive symptoms [31].

Social support, which can be understood as the material and emotional resources provided to an individual through interpersonal interactions [32], is known to be a protective factor in pregnancy in terms of preterm birth [33], low birth weight [34] and perinatal depression [35, 36]. However, many women who have emigrated find themselves without the support of their family and community [11], placing them at an even greater risk of not having access to resources to help meet the demands of pregnancy [37]. Findings from a qualitative study conducted in Toronto, Canada, identified lack of proximity to informal support systems (family) and barriers to formal support (community services) due to lack of knowledge or language proficiency as contributing to postpartum depressive symptoms [12].

Systemic challenges also increase the levels of psychosocial stress of ethnic minorities during pregnancy [16, 38]. Women who have immigrated, and may have language barriers, often face systemic barriers that limit access to resources or opportunities in society such as employment, education, and health care. For example, policies that prevent educational credentials from being recognized create both educational and employment barriers. Accessing primary care may also be a challenge if services are not offered in a culturally sensitive way. For example, researchers Reitmanova and Gutafson [39] identified systemic barriers that women who identified as Islamic faced when accessing prenatal care. Specifically, lack of culturally and linguistically appropriate information was identified as a barrier to accessing maternal health services. They also found that women in this subpopulation experienced discrimination and cultural insensitivity from service providers. In addition to occurences of cultural insensitivity, women from ethnic minority groups may experience compounding effects of racial discrimination. Racism contributes to social isolation during pregnancy [33] and throughout motherhood [40]. Institutionalized racism against minority status women has perpetuated the structural barriers that prevent women from accessing medical, psychological and financial resources [8].

Despite the known physical, mental, and social consequences of elevated stress in pregnancy, little research has been devoted to identifying the specific psychosocial outcomes of mothers from an ethnic minority in Canada. Most research has used public health records that do not include psychosocial measures and tend to dichotomize populations into White and non-White categories. The few Canadian population studies that have disaggregated immigrant populations *have* found significant differences in health outcomes by place of origin [41]. However, to our knowledge, there are no existing comparison studies that have explored the specific types of psychosocial stressors experienced by Canadian women who identify with an ethnic minority. The aim of this study was to identify specific types of psychosocial stressors experienced by Canadian women who identified with an ethnic minority. Specifically, when compared to women of dominant culture (White), do women who identify with an ethnic minority (Asian, Arab, Other Asian, African, First Nations and Latin American) experience greater levels of psychosocial stress during pregnancy?

## Methods

In this comparative descriptive study, a secondary analysis was conducted of data collected for the community-based, All Our Babies (AOB) prospective pregnancy cohort in Calgary, Canada. The AOB study was designed to explore the environmental and genetic risk factors for preterm birth [42]. An ethics review board approved the study (E ID 22128). Calgary is one of the most ethnically diverse cities in Canada. Ethnic minorities comprise 28 % of the total population, with the three most populous minority groups in Calgary being South Asian, Chinese, and Filipino [43]. Calgary also has the fourth highest immigrant population in Canada representing 26 % of its total population. Calgary depends on immigration to sustain its rapidly growing economy [44] and in 2012, Calgary became home to 70,700 newcomers to Canada [43].

Between May 2008 and December 2010, a convenience sample of participants (*N* = 3,552) was recruited through primary health care clinics (*n* = 573), community posters and word of mouth (*n* = 675), and Calgary Laboratory Services (*n* = 2,763). Eligible participants were < 25 weeks gestation at recruitment, 18 years of age or older, receiving prenatal care in Calgary, and sufficiently proficient in English to complete the questionnaires. Women were asked to describe their ethnic background by selecting from a list [45]. For the purpose of this study, particpants who identified with White/Caucasian group were considered the *dominant culture*, due to the fact that the dominant culture in Canada has been historically influenced by European cultures that have dominated social, political, and economic institutions [46]. To assess psychosocial stress, several scales recognized for their continued use and validity in assessing psychosocial stress in pregnancy were included (see Table 1). All inter-correlations among the different psychosocial stress measures were significant at *p* < .001.

**Table 1:** Description of variables and measures

Variable/measure	Gestation collected	Description
Ethnicity	<25 weeks	Ethnicity coded as White, Black/African, North American, First Nations (that included registered, unregistered, and Metis), Asian (including Chinese, Japanese and Korean), Other Asian (including South Asian, Filipino, Southeast Asian, and West Asian), Arab, Latin American, and Mixed/Other. Dominant culture = White/Caucasian; ethnic minority = all other ethnicities.
Edinburgh Postnatal Depression Scale (EPDS) [20, 73]	<25 and 34–36 weeks	10-item self-report scale to measure depressive symptoms in pregnant and postnatal women. Excludes somatic symptoms such as fatigue and change in appetite as these changes are likely to occur in pregnancy and could possibly confound the scores. Theoretical range 3 to 30; response categories vary by item; scores of ≥ 13 or greater are considered problematic. Sensitivity = .86; specificity = .78. EPDS discriminates between women with a diagnosis of depression and those without. A score ≥13 at either time point was defined as depressive symptoms.
MOS Short Form Health Survey-12 (SF-12) [74]	<25 and 34–36 weeks	12-item self-report scale adapted from the Medical Outcomes Study 36-Item health survey [74] to measure eight health concepts: physical functioning, physical role, bodily pain, general health; vitality, social functioning, emotional role, and mental health. Response categories vary by item., Temporal stability over 2 weeks = .89 for physical and .76 for mental components. The SF-12 discriminates between patients with minor and serious physical and mental conditions. Concurrent validity with SF-36 *r* = .95. SF-12 was computer-scored and coded as above or below the developers’ cut-off.
Medical Outcomes Study (MOS) Social Support Survey (SSS) [75]	<25 and 34–36 weeks	19-item self-report scale to measure perception of the availability of different forms of support: emotional support, tangible/functional support, informational support, and positive social interactions. Used extensively to explore the interaction of social support and health outcomes [76]. Theoretical range of scores is 19 to 100; higher scores indicate greater social support. Items are summed to create subscale and overall social support index. A score ≤ 69 at either time point was defined as inadequate social support.
Perceived Stress Scale (PSS) [77]	<25 and 34–36 weeks	Widely used [78], 10-item self-report scale to measure perceived stress related to unpredictability, uncontrollability, and overload over the last month. Theoretical range of scores is 10 to 50; higher score indicates higher stress. Cronbach’s α = .85; temporal stability over 2 days = .85. For this study, a PSS score in the top 20^th^ percentile at either time point was considered problematic.
State-Trait Anxiety Inventory (STAI) [79]	<25 and 34–36 weeks	20-item, self-report measure of anxiety in adults on two dimensions: State (temporary condition) and Trait (general and long-standing quality). Theoretical range of scores is 20 to 80; higher score indicates higher anxiety. For this study, only State Anxiety scores were used. Scores ≥ 40 at either time point indicated the presence of State Anxiety.

### Procedures

Questionnaires were sent by mail to the participant’s home at < 25 weeks gestation, and between 34 and 36 weeks gestation. Of the 4,011 questionnaire packages sent, 3,552 mothers completed at least one survey (89 % response rate), and 123 discontinued after the first survey. Eight women were deemed ineligible due to language barriers.

Data were examined for missing values as not all participants had complete data sets. To understand the differences between complete and incomplete data, we compared missing and complete values for each variable of interest. Women whose first language was English or French were more likely to have completed all the measures of interest than those for whom English or French was not their first language, *χ*^2^ (2, *N* = 3,359) = 7.41, *p* < .01. There were significant differences in missing and non-missing health outcomes among ethnic groups, *χ*^2^ (6, *N* = 3,354) = 30.02, *p* < .01. First Nations participants had the most missing data (31 %), followed by Other Asian, Arab, and Latin American (20 %), Mixed/Other (19 %), Black (14 %), while White (7.6 %) and Asian (7 %) had the least missing data.

### Data analysis

Analyses consisted of descriptive statistics (means, frequencies/percentages) for ethnicity, income, marital status, parity, and age. Standardized cut-off scores were used to assess psychological distress. Psychosocial measures were compared by ethnicity using chi-square tests for statistical independence. To determine if there was an independent association between household income and psychosocial stress, measures were compared across the following household income levels: <$20,000, $20,000 to < $40,000, $40,000 to < $70,000, $70,000 to < $100,000 and > $100,000. Analyses were performed using SPSS version 22 with alpha set at 0.05 level.

## Results

Demographic and health characteristics of mothers presented in Table 2 show that the percentage of mothers who identified with an ethnic minority (21 %) was similar to the percentage of percentage of Canadians who identify as an ethnic minority (19.1 %) [43]. A sub-analysis of birthplace and ethnic identity revealed that 91 % of women who identified with an ethnic minority had either immigrated to Canada or were first generation Canadians; 1 % were of Aboriginal heritage and the remaining 8 % were > than first generation Canadians.

**Table 2:** Demographic characteristics of sample

Characteristic	*n* (%)
Maternal Age (*n* = 3283)	
18–24	296 (9.0)
25–29	1018 (31.0)
30–34	1327 (40.4)
35–39	552 (17.1)
40+	80 (2.4)
Marital Status (*n* = 3354)	
Married/Common Law	3165 (94.4)
Other	189 (5.6)
Education (*n* = 3356)	
High School or Less	370 (11.0)
Some or completed university/college	2458 (73.2)
Some or completed graduate school	528 (15.7)
Income (*n* = 3252)	
< $20,000	105 (3.0)
$20,000–40,000	194 (5.5)
$40,000–70,000	484 (13.6)
$70,000–99,999	797 (22.4)
≥ 100,000	1672 (47.1)
Language (*n* = 3359)	
English	2967 (83.5)
French	23 (0.6)
Other	369 (10.4)
Ethnic Identity (*n* = 3552)	
White	2636 (74.2)
Black/African	50 (1.4)
First Nations	32 (0.9)
Asian	164 (4.6)
Other Asian	232 (6.5)
Arab	43 (1.2)
Latin American	79 (2.2)
Mixed/Other	118 (3.3)
Did Not Answer	198 (5.6)

For each measure of psychosocial stress, women of ethnic minorities were aggregated and compared to the White reference group using chi square test for independence between measures of psychosocial stress and ethnicity (Table 3). Women of an ethnic minority were nearly three times as likely to report inadequate social support (28.8 %) than the white reference group (9.8 %). Depressive symptoms were more prevalent amongst the ethnic minority group (9.8 %) than amongst the White reference group (5.9 %). Women of an ethnic minority reported more symptoms of anxiety (22.9 %) than the White reference group (17.0 %) and percieved their life as more stressful (28.9 % to 19.7 % respectively). In measures of physical health, more women of an ethnic minority reported poorer physical health (21.4 %) than the White reference group (14.1 %). More women of an ethnic minority group reported poor emotional health over the course of their pregnancy (16.9 %) than the White reference group (10.4 %).

**Table 3:** Comparative analysis of measures between the ethnic minority aggregate and the White reference group

	White reference group		Aggregate of ethnic minorities		
	Symptomatic^a^ *n* (%)	Normal *n* (%)	Symptomatic *n* (%)	Normal *n* (%)	*X* ^2^ (df = 1)
Social Support (*N* = 3318)	256 (9.8)	2363 (90.2)	201 (28.8)	498 (71.2)	167.370*
Depression (*N* = 3141)	148 (5.9)	2342 (94.1)	64 (9.8)	587 (90.2)	12.390*
State Anxiety (*N* = 3080)	420 (17.0)	2044 (83)	141 (22.9)	475 (77.1)	11.299*
PSS^b^ at <25 weeks (*N* = 3311)	513 (19.7)	2093 (80.3)	204 (28.9)	501 (71.1)	27.989*
PSS at 35 (±1) weeks (*N* = 2899)	489 (21.2)	1813 (78.8)	168 (28.1)	429 (71.9)	12.871*
Emotional Health (*N* = 3353)	275 (10.4)	2360 (89.6)	121 (16.9)	597 (83.1)	22.300*
Physical Health (*N* = 3353)	371 (14.1)	2264 (85.9)	154 (21.4)	564 (78.6)	23.200*

*p ≤ .001

^a^Symptomatic = above clinical cut-off scores

^b^ *PSS* perceived stress scale

The chi square analysis of disaggregated ethnic groups (i.e., Asian, Arab, Other Asian, African, First Nations, Latin American, Mixed/Other, or White; Table 4) showed women of an ethnic minority more often reported elevated levels of inadequate social support, anxiety, percieved stress, as well as poorer physical and emotional health compared to the White reference group. Over a third of the Arab (35.7 %) and Other Asian (33.9 %) group reported having inadequate social support and over a quarter of all Black/African American (28.6 %), Latin American (28.2 %), First Nations (28.1 %), and Asian (27.5 %), while only 9.8 % of the white reference group reported inadequate support. White women were least likely to report symptoms of depression (5.9 %) while over twice as many Arab (15.4 %) and Other Asian (12.9 %) reported depressive symptoms.

**Table 4:** Comparative analysis of measures between ethnic minorities and the White reference group

	Ethnicity								
	White	Asian	Other Asian	Arab	Latin American	Black	First Nations	Mixed/other	
	n (%)	n (%)	n (%)	n (%)	n (%)	n (%)	n (%)	n (%)	*X* ^2^ (df = 7)
Social Support (*N* = 3318)									
Symptomatic^a^	256 (9.8)	44 (27.5)	76 (33.9)	15 (35.7)	22 (28.2)	14 (28.6)	9 (28.1)	21 (18.4)	184.626*
Normal	2363 (90.2)	116 (72.5)	148 (66.1)	27 (64.3)	56 (71.8)	35 (71.4)	23 (71.9)	93 (81.6)	
Depression (*N* = 3141)									
Symptomatic	148 (5.9)	11 (7.0)	27 (12.9)	6 (15.4)	5 (7.2)	3 (6.8)	2 (7.7)	10 (9.4)	21.116*
Normal	2342 (94.1)	147 (93.0)	182 (87.1)	33 (84.6)	64 (92.8)	41 (93.2)	24 (92.3)	96 (90.6)	
State Anxiety (*N* = 3080)									
Symptomatic	420 (17.0)	24 (16.0)	44 (22.6)	17 (50.0)	18 (27.7)	11 (26.2)	7 (29.2)	20 (18.9)	35.966*
Normal	2044 (83.0)	126 (84.0)	151 (77.4)	17 (50.0)	47 (72.3)	31 (73.8)	17 (70.8)	86 (81.1)	
PSS^b^ at <25 weeks (*N* = 3311)									
Symptomatic	513 (19.7)	39 (24.1)	74 (32.9)	17 (39.5)	22 (28.9)	14 (28.6)	12 (37.5)	26 (22.0)	39.867*
Normal	2093 (80.3)	123 (75.9)	151 (67.1)	26 (60.5)	54 (71.1)	35 (71.4)	20 (62.5)	92 (78.0)	
PSS at 35 (±1) weeks (*N* = 2899)									
Symptomatic	489 (21.2)	30 (21.4)	63 (31.8)	16 (44.4)	15 (23.1)	11 (26.2)	9 (45.0)	24 (25.0)	28.283*
Normal	1813 (78.8)	110 (78.6)	135 (68.2)	20 (55.6)	50 (76.9)	31 (73.8)	11 (55.0)	72 (75.0)	
Emotional Health (*N* = 3353)									
Symptomatic	275 (10.4)	29 (17.7)	43 (18.5)	8 (18.6)	9 (11.4)	10 (20.0)	9 (28.1)	13 (11)	33.665*
Normal	2360 (89.6)	135 (82.3)	189 (81.5)	35 (81.4)	70 (88.6)	40 (80.0)	23 (71.9)	105 (89)	
Physical Health (*N* = 3353)									
Symptomatic	371 (14.1)	39 (23.8)	57 (24.6)	6 (14.0)	13 (16.5)	7 (14.0)	9 (28.1)	23 (19.5)	32.429*
Normal	2264 (85.9)	125 (76.2)	175 (75.4)	37 (86.0)	66 (83.5)	43 (86.0)	23 (71.9)	95 (80.5)	

*p ≤ .005

^a^Symptomatic = above clinical cut-off scores

^b^ *PSS* perceived stress scale

State anxiety was reported by half of all Arab women, while only 17 % of the White reference group reported symptoms of anxiety. Arab women were also the most likely to percieve their life situation as stressful (39.5 %) at < 25 weeks gestation. Arab and First Nations women were twice as likely to percieve their life situation as stressful (44.4 and 45 % respectively) than the White reference group (21.2 %) at 34 to 36 weeks gestation. In measures of physical health, the First Nations (28.1 %), Other Asian (24.6 %), and Asian (23.8 %) groups reported the poorest perceptions of physical health.

### Income

There was an association between income levels and measures of social support *χ*^2^ (4, *N* = 3,326) = 207.3, *p* < .01, state anxiety *χ*^2^ (4, *N* = 3109) = 62.8, *p* < .01, depression *χ*^2^ (4, *N* = 3,172) = 29.8, *p* < .01, and perceived stress *χ*^2^ (4, *N* = 3,319) = 166, *p* < .01.

## Discussion

Women who identified with an ethnic minority experienced greater psychosocial stress during pregnancy than women from the White reference group. While the aggregate of ethnic minority groups more frequently reported poor outcomes in measures of social, emotional, and physical health, the disaggregation of ethnic groups identified specific sub-populations that were at greater risk for poor mental health outcomes.

### Social support

More women of an ethnic minority reported lower levels of social support compared to women from the White reference group. Curiously, the vast majority (>90 %) of women from the White reference group (used as a proxy for Western/individualistic orientation) reported adequate levels of social support. Although collectivist and individualist cultures differ in the extent to which cooperation, competition, or individualism are emphasized [47], inadequate social support may not be a function of the culture of origin [48]. In other words, it should not be assumed that women who identify with individualistic cultures experience lower levels of social support, nor that women from collectivist cultures experience greater levels of social support. In the present study, the issue of inadequate support may be a function of immigration and/or minority status and not Canadian culture, as the majority of women from Western/individualistic cultural orientation report experiencing adequate support. Alternatively, it could be the adequacy of social support in an western individualist society is inadequate for those who immigrate from collectivist cultures.

In this study, approximately 70 % of participants who identified with an ethnic minority were foreign-born. Perhaps the stress of immigration is a contributing factor to the elevated levels of psychosocial stress. Women who have migrated from another country often find themselves without the support of their family and community [11]. Falicov [49] argues that inherent with migration is a loss of social capital. This may be especially true for women who have migrated from collectivist cultures such as Asia, Africa, South Asia, and Latin America, where greater levels of familial and culturally relevant support are embedded in the society and this level of support may not be established in western society [50].

In the present study, over a third of Arab and Other/Asian participants reported inadequate levels of social support. Witt and colleagues [51] argue that it should not be assumed that women who identify with Arab or Other Asian ethnicity experience lower levels of social support as a function of Arab culture. Many of the countries that were categorized into the Other Asian group could also be identified as Arab countries [52]; thus, there may be overlap between the two cultural identities. According to Kridli [52], pregnancy is highly valued in Arab culture, and pregnant women typically receive considerable support from their husband and family throughout pregnancy. In their recent population-based study exploring levels of social support amongst Iranian women, [32] the majority of women reported levels of social support similar to their Western counterparts. However, women in the present study who identified with Arab or Other Asian ethnicity reported the highest levels of *inadequate* support. Rather than inadequate support being a function of ethnic identity (i.e., that Arab women receive less social support) or Canadian culture, it is likely that many of the women in the present study who migrated to Canada lost the proximal support of their extended family.

Over a quarter of women of Latin American ethnicity reported inadequate levels of social support. The collectivist orientation of Latin culture is especially influenced by *familism*, which places extended family at the centre of social life [53]. In Latin American cultures, the extended family is typically the primary source of social support. Again, inadequate social support amongst the Latin American women may be a result of migration away from their extended family. Similarly, women identifying with African ethnicity were more likely to report inadequate social support. According to Stewart [54], African cultures generally expect that close friends and extended family will provide emotional and tangible support to help pregnant and new mothers whenever possible. In many African countries, pregnancy is highly celebrated and supported [55]. During and after birth, a woman’s mother assumes responsibility of looking after her daughter and newborn grandchild. These practices and beliefs about motherhood are considerably different from those of western cultures, and may contribute to a perception of low social support relative to support expectations in her country of origin.

Systemic barriers may also contribute to difficulties in accessing social supports. Reitmanova and Gustafson [39] identified several barriers to accessing culturally responsive maternal health care such as language barriers, lack of flexibility for cultural practices, and having men present at the prenatal classes (not acceptable in all cultures/religions). In a qualitative study conducted by Ahmed and colleagues [12], barriers to accessing community supports included lack of information in preferred language and lack of culturally competent primary care providers. Despite dependence on immigration for population growth, maternal care programs continue to be Eurocentrically organized and unresponsive to Canada’s growing cultural diversity [12].

In addition to the above systemic barriers to accessing social support, there may be differences in support-seeking behaviours among groups. In a study of support-seeking attitudes, researchers Kim and colleagues [50] found that participants who identified as Asian or a subgroup of Asia (e.g., Vietnamese, Filipino, Korean) were less likely to seek support than European Americans. Seeking help was associated with negative self-stigma and a reduced sense of self-efficacy for Asian-Americans. In the current study, over a quarter of the women who identified as Asian reported having inadequate levels of social support. If seeking social support in pregnancy is not considered a culturally appropriate behaviour, inadequate levels of social support may be a function of help-seeking attitudes.

### Depression

The overall prevalence of depressive symptoms in the current study were on par with the estimated 1-year prevalence of depression amongst Canadian adult women, at 6–7 % [56]. In contrast, other studies found higher rates of depressive symptoms amongst pregnant women. In a population-based study that utilized the EPDS, the reported the prevalence rate of depressive symptoms in the last trimester of pregnancy was 17 % [57]. A systematic review of depression during pregnancy by Bennet and colleagues [58] reported the overall rates of women detected as having depressive disorder at 12.8 % during the second trimester and 12.0 % during the third trimester. In comparison with prevalence rates of depressive symptoms in similar population studies using the EDPS, the women in this study reported fewer depressive symptoms.

When using depressive symptoms as a measure, the hypothesis that women of an ethnic minority have greater levels of psychosocial stress during pregnancy was supported in this study: All ethnic minority groups reported higher levels of depressive symptoms during pregnancy than the White reference group. This contrasts with results from the Canadian Community Health Survey that found minority groups and immigrant populations reported fewer depressive symptoms than non-immigrant populations [59].

Findings from the present study are consistent with other recent research on minority status and depressive symptoms. For example, a recent population-based study of 23 European countries reported that immigrants and ethnic minorities experienced more depressive symptoms than those from the dominant culture [60]. Research conducted in a working population in Germany found that migration status may also contribute to depressive symptoms in that migrant females were twice as likely to report depressive symptoms as non-migrant females [61].

### Anxiety

The overall prevalence of anxiety symptoms in the current study was three times higher (18 %) than the Canadian 1-year estimated prevalence rates of 6 % amongst females [62]. In the current study, perhaps the most surprising finding was that symptoms of anxiety were reported by half of all women who identified with Arab ethnicity. In a large-scale population study conducted in Lebanon, which is a member state of the Arab League, Karam and colleagues [63] reported a 12-month prevalence of anxiety disorders to be 11.2 %. In a smaller cross sectional study (*N* = 800) conducted in Morocco (also a member state of the Arab League), 25.5 % of the participants met the criteria for an anxiety disorder [64]. Although higher rates of anxiety disorders were reported in the second study, twice as many women who identified as Arab in the present study reported symptoms of anxiety, suggesting that this Arab women may face additional challenges in the Canadian context, which warrants further exploration.

### Perceived stress

In the second trimester of pregnancy, more women of an ethnic minority than those in the White reference group perceived their life situation as stressful. Arab women reported the highest levels of perceived stress in the second trimester, and an even greater percentage of Arab women perceived their life situation as stressful in the third trimester. Women who identified as First Nations, Other Asian, Latin American, Black, and Asian also reported higher levels of perceived stress in the second and third trimester of pregnancy than the White White reference group. As with women who identified as Arab, there was an increase in the perception of stress with advancing gestation. Glynn and colleagues [25] examined whether women who do not experience a decline in perceived stress with advancing gestation may be at greater risk to deliver preterm. Indeed, they found a modest correlation between preterm birth and increased perceived stress and state anxiety in mid- to late- gestation.

### Emotional health

The vast majority (88 %) of women in this study rated their emotional health as good. Interestingly, poor emotional health was reported less frequently than perceived stress, anxiety, or inadequate social support, all indicators of poor emotional health [35, 65–68]. The emotional health reported in the current study suggests that there is a difference between objective measures of mental health (e.g., SAI, EPDS) and the subjective interpretations of emotional health. This anomaly has been observed elsewhere [69].

Despite the high percentage of good emotional health reported, the overall percentage of women reporting poor emotional health was greater than has been found in previous studies. In a population-based study of pregnant women conducted by Witt and colleagues [51] 8 % of women reported their mental health as poor, which is less than the 11 % reported in the current study. Women who identified with an ethnic minority were twice as likely to report poor emotional health than the White reference group.

### Physical health

As with emotional health, the majority (84 %) of women in this study reported being in good physical health; however, more women of an ethnic minority reported their health as poor than the White reference group. In a secondary analysis of the Canadian Community Health survey, Romans and colleagues [70] found that only 9 % of individuals living in Canadian urban centers rated their physical health as poor. Considering the physical demands of pregnancy, it is difficult to know if poor physical health reports were related to pregnancy or underlying health problems that may be exacerbated with pregnancy.

Interestingly, women who identified as Arab, Black/African, or White reported the same levels of good physical health throughout pregnancy. As with emotional health, First Nations women were the least likely to report being in good physical health. This finding is consistent with the small body of literature that addresses physical health disparities amongst First Nations Women. Across Canada, First Nations women have poorer physical health trajectories than all other Canadians [71, 72]. Unfortunately, pregnancy has been shown to further increase the risk for developing metabolic problems such as gestational diabetes in First Nations women [1].

### Summary of psychosocial stress measures

In every psychosocial measure analyzed, more women who identified with an ethnic minority had elevated symptoms of problems compared to the White reference group. By further separating the analyses into subpopulations, important group differences were identified. Although the intent of this analysis was to measure psychosocial stress, the use of mental health screening instruments also identified women at greatest risk for mental health problems. Findings from this study are important to consider in relation to primary care. Specifically, it is important for mental health care professionals to recognize that women who identify with an ethnic minority may require additional supports to reduce the risk for poor mental health.

### Limitations

The current research is an attempt to explore psychological distress during pregnancy, but it is part of a much larger interdisciplinary project. The inventories were selected based on the goals of the larger project and were not necessarily the best available measures to answer the research question.

Ethnic identity was determined by having participants choose from a list of 15 ethnic categories. However, in Canada, there are more than 200 different ethnic origins represented [45]. Thus, categories were in no way exhaustive nor able to capture the extent of Canadian diversity. From the categories selected, ethnicity was further collapsed into seven groups. Although the groups were chosen according to historical, geographic and cultural similarities, there is a tremendous amount of ethnic diversity within each group. Although frequently used cross culturally, not all measures reported psychometric properties for cross-cultural validity. Characteristics of participants in this study may have been more informative if the data were collected at interval level.

It is possible that this study may not have captured the most vulnerable populations. The inclusion criteria for participation in the AOB study required participants to be proficient in English and receiving prenatal care. Women who were not receiving prenatal care, transient, and/or who experience language barriers may be at a higher risk for poor physical and mental health outcomes. Furthermore, even within the AOB sample, the most vulnerable women may be under represented due to incomplete data sets. Although the AOB sample is representative of Canadians in fertility age, ethnic distribution, and level of education, their economic status was higher than national averages. As there was an association between income levels and psychosocial measures, future anyalysis may include household income as a mediator of psychosocial stress. Also, pregnancy care programs and other community supports differ relative to the municipality. Thus, caution should be used when generalizing these findings to other populations outside of Calgary.

## Conclusions

Women who identify with an ethnic minority face all the typical challenges of motherhood in addition to dealing with the complexities of acculturation, language barriers, shifting role expectations, increased vulnerabilities, and vocational barriers. Findings from the present study have identified the need for further exploration into the unique experiences of women who identify with an ethnic minority. Although this study identified that higher proportions of ethnic minorities compared to the White reference group experienced poor psychosocial outcomes, further research is needed to understand specific contributing factors. For example, it may be that satisfactory social support in an individualistic culture is insufficient support in a collectivist culture, and that the inadequacy of support combined with the challenges associated with membership in the non-dominant culture significantly elevates the risk for poor outcomes. Further qualitative exploration may help elucidate these findings.

The current study provides a unique perspective into risk factors and mental health trends of a Canadian urban population. Relating back to Canada’s immigration policy, Canada relies on its growing immigrant population for the growth and vitality of our nation. As such, it is in the best interest of Canada to help ensure optimal health and well-being of its citizens, including those who are often least empowered and most vulnerable: women of an ethnic minority.

## Abbreviations

AOB, all our babies; SAI, state anxiety index; EPDS, Edinburgh postnatal depression scale; PSS, perceived stress scale; SF-12, short form health survey-12; MOS, medical outcomes survey of social support; PPD, postpartum depression
